# *Aedes* Mosquito Saliva Modulates Rift Valley Fever Virus Pathogenicity

**DOI:** 10.1371/journal.pntd.0002237

**Published:** 2013-06-13

**Authors:** Alain Le Coupanec, Divya Babin, Laurence Fiette, Grégory Jouvion, Patrick Ave, Dorothee Misse, Michèle Bouloy, Valerie Choumet

**Affiliations:** 1 Unité de Génétique Moléculaire des Bunyavirus, Institut Pasteur, Paris, France; 2 Unité d'Histopathologie humaine et modèles animaux, Institut Pasteur, Paris, France; 3 MIVEGEC (IRD 224 CNRS 5290-UM1-UM2) Maladies infectieuses et vecteurs: écologie, génétique, évolution et contrôle, Centre IRD de Montpellier, Montpellier, France; United States Army Medical Research Institute of Infectious Diseases, United States of America

## Abstract

**Background:**

Rift Valley fever (RVF) is a severe mosquito-borne disease affecting humans and domestic ruminants. Mosquito saliva contains compounds that counteract the hemostatic, inflammatory, and immune responses of the host. Modulation of these defensive responses may facilitate virus infection. Indeed, *Aedes* mosquito saliva played a crucial role in the vector's capacity to effectively transfer arboviruses such as the Cache Valley and West Nile viruses. The role of mosquito saliva in the transmission of Rift Valley fever virus (RVFV) has not been investigated.

**Objective:**

Using a murine model, we explored the potential for mosquitoes to impact the course of RVF disease by determining whether differences in pathogenesis occurred in the presence or absence of mosquito saliva and salivary gland extract.

**Methods:**

C57BL/6NRJ male mice were infected with the ZH548 strain of RVFV via intraperitoneal or intradermal route, or via bites from RVFV-exposed mosquitoes. The virus titers in mosquitoes and mouse organs were determined by plaque assays.

**Findings:**

After intraperitoneal injection, RVFV infection primarily resulted in liver damage. In contrast, RVFV infection via intradermal injection caused both liver and neurological symptoms and this route best mimicked the natural infection by mosquitoes. Co-injections of RVFV with salivary gland extract or saliva via intradermal route increased the mortality rates of mice, as well as the virus titers measured in several organs and in the blood. Furthermore, the blood cell counts of infected mice were altered compared to those of uninfected mice.

**Interpretation:**

Different routes of infection determine the pattern in which the virus spreads and the organs it targets. *Aedes* saliva significantly increases the pathogenicity of RVFV.

## Author Summary

Rift Valley fever is an endemic and epidemic zoonosis in Africa and the Arabic Peninsula. In humans, in the most severe cases the viral infection causes fulminant hepatitis associated with haemorrhagic fever, permanent blindness or severe encephalitis. Despite the importance of vector transmission in the spread of arboviruses, few studies on the physiopathology of viral infection have considered the role of the arthropod in the efficiency of viral infection. Moreover, the route of virus inoculation and the presence of the vector's saliva can potentially affect virus pathogenicity. Our results show that saliva from *Aedes* mosquitoes increases Rift Valley fever pathogenicity. Importantly, our study also revealed that RVFV transmitted *via* mosquito bites spread differently than virus inoculated by other routes. These observations may have interesting repercussions given the role mosquitoes were shown to play in the transmission of RVFV in humans during the last outbreak of the disease in Saudi Arabia. Identification of salivary proteins able to increase RVFV virulence may pave the way to new approaches to prevent or cure the disease.

## Introduction

Rift Valley fever virus (RVFV) is a zoonotic mosquito-borne virus which causes epizootics and associated human epidemics throughout Africa [1], [2]. First identified in Kenya in 1931 [3], RVFV is now considered an endemic zoonotic agent in sub-Saharan Africa causing explosive outbreaks in animals and humans. It has been observed in Egypt, Mauritania, and the Arabic Peninsula [4], [5], [6]. The manifestation of severe RVF disease in humans is variable. Humans may develop a wide range of clinical signs including hepatitis, retinitis, and delayed-onset encephalitis and, in the most severe cases, haemorrhagic disease. The overall case fatality ratio is estimated to be between 0.5% and 2% [7], [8], [9]. In Yemen and Saudi Arabia, a RVFV outbreak resulted in approximately 2,000 human infections and 250 deaths (CDC 2000). A study of the RVFV epidemic in Saudi Arabia reported a high incidence of neurological manifestations (17.1%) in infected individuals [7]. Mosquito bites were reported to play an important role in the transmission of the disease during this outbreak.

RVFV belongs to the genus *Phlebovirus* in the family *Bunyaviridae*. Its tripartite negative-strand RNA genome is composed of a large segment (L) that encodes the L protein, which is the viral RNA-dependent RNA polymerase; a medium segment (M) that encodes a single open reading frame (ORF) generating the NSm, G1 (Gc) and G2 (Gn) proteins and a small segment (S) that encodes the nucleocapsid protein (N) and a nonstructural protein (NSs) using an ambisense strategy [10]. NSs was shown to suppress interferon induction (Billecocq et al., 2004).

RVFV can be transmitted to vertebrates by several species of mosquitoes such as *Aedes* spp. and *Culex* spp. Human infections typically occur through bites from infected mosquitoes, through percutaneous/aerosol exposure during the slaughter of infected animals, or via contact with aborted fetal materials. Transmission efficiency depends on the ability of the virus to cross the various barriers in the vector [11]. Therefore, after a mosquito takes a blood meal from an infected individual, the ingested virus passes into the midgut of the mosquito where it replicates before infecting different organs in the mosquito. At the end of the extrinsic incubation period in the vector, salivary glands are infected and the virus is transmitted by saliva during a blood meal. The reproductive system of the mosquito is also infected and transovarial transmission is important for long term maintenance of the virus [12]. Worldwide RVFV is considered as a potential biological weapon. Both modified live attenuated virus and inactivated virus vaccines have been developed for veterinary use, but there are currently no commercially available vaccines for humans.

During a blood meal, insects are subject to defensive responses from the vertebrate, including hemostasis and the immune response. In this context, the saliva injected by the mosquito plays multiple roles. Indeed, saliva proteins have angiogenic, anti-hemostatic, anti-inflammatory and immunomodulatory properties [13]. The various properties of the saliva proteins towards the host immune response affect the pathogen transmission. In some cases, co-injection of virus and saliva potentiates viral infection of the vertebrate [14], [15], [16], [17]. In other cases, pre-exposure to saliva generates enhances mortality from subsequent viral infection via mosquito bite [18]. A longer viremia was observed in deer and chipmunks infected by mosquito bite containing La Crosse virus, another member of the *Bunyaviridae* family, compared to syringe injection [19]. Potentiation of infection by mosquito saliva was also demonstrated for Cache Valley virus, an orthobunyavirus that also belongs to the *Bunyaviridae* family [14]. These observations raise the question of whether RVFV infection is also potentiated by mosquito saliva. Since RVFV is also transmitted by blood and aerosols, the context for its transmission differs from those of other viruses studied previously. In this project, our objective was to evaluate the role of *Aedes* mosquito saliva in the natural transmission of RVFV. For this purpose, we make use of an animal model that allowed us to study the pathogenesis of RVFV infection. We evaluated two different routes of infection: the intraperitoneal route, which has been utilized in most previous studies of RVFV pathogenesis, and the intradermal route, which mimics the mosquito bite. We also used non-infected and RVFV-infected mosquitoes to evaluate the role of saliva in the progression of the disease. Importantly, we found that *Aedes* saliva potentiated RVFV infection, once again highlighting its role in arbovirus transmission.

## Materials and Methods

### Ethic statement

All studies on animals followed the guidelines on the ethical use of animals from the European Communities Council Directive of November 24, 1986 (86/609/EEC). All animal experiments were approved and conducted in accordance with the Institut Pasteur Biosafety Committee. Animals were housed in the Institut Pasteur animal facilities accredited by the French Ministry of Agriculture to perform experiments on live mice, in appliance of the French and European regulations on care and protection of the Laboratory Animals (accreditation number B 75 15-01 and B 75 15-07). The study protocols were approved by the Comité d'Ethique pour l'Expérimentation Animale (CEEA) - Ile de France - Paris - Comité 1.

The ZH548 strain was isolated from a human infection during the 1977 outbreak in Egypt [18]. The case was anonymous and an informed consent was not required at that time. This strain was part of a collection used by the NRC of arboviruses (B. Le Guenno and H. Zeller). This collection was transmitted to us and we possess an AFSSA authorization of detention, transfer and manipulation (since 2001) as a “select agent”.

### Mice, virus and cells

We used the DBA-1 and C57BL/6-NRJ mice for infections (Janvier, France). Vero E6 cells were grown in DMEM supplemented with 10% fetal bovine serum (FBS), 10 µg/ml of penicillin and 10 U/ml of streptomycin. C6/36 cells were grown at 28°C in plastic cell culture flasks in Leibovitz medium 15 supplemented with 10% FBS, penicillin (50 units/m1), and streptomycin (50 mg/ml).

Stocks of the virulent Egyptian ZH548 RVFV strain were produced under biosafety level 3 (BSL3) conditions. In all experiments, the ZH548 strain was obtained from a cell culture of C6/36 cells. It was produced under BSL3 conditions.

### Mosquitoes

Dehydrated eggs of *Aedes aegypti* (strain PAEA) and *Ae.vexans vexans* were placed in water to hatch. Adult mosquitoes were reared in a room held at 25±1°C and 80% relative humidity, and having a light/dark ratio of 12 h/12 h. The larvae were fed on brewer's yeast tablets and adults were fed on sugar water (10%).

### Mosquito infection by the ZH548 RVFV strain

Rabbit blood was collected in heparinized tubes (0.02%). Red blood cells were separated from plasma by centrifugation, washed 3 times in 1X PBS, and were resuspended in the same buffer. Five-day old female mosquitoes were placed in boxes sealed with veils and were fed on 37°C thermostated glass feeders covered with chicken skin and filled with a mixture containing 2 mL of red cells, 1 mL of virus solution (10^8^ plaque forming unit (pfu)/mL) and 30 µL of ATP (5.10^−3^ M).

### Preparation of salivary gland extracts

Mosquito females were blood-fed five days after hatching. Three weeks later (corresponding to the extrinsic incubation period of RVFV in *Ae. aegypti* and *Ae. vexans* mosquitoes), 100 salivary glands (SG) were dissected and placed in 100 µL 1X PBS. The inocula used in our experiments were equivalent to a pair of SG (or two salivary glands extracts [SGE]). SG-containing tubes were stored at −80°C. SGEs were prepared by sonicating the SGs (five times at 4 min each with a pulse ratio of 2 sec on/2 sec off) and centrifuging the crude extract at 13,000 rpm for 15 min at 4°C. The supernatant was transferred to clean tubes and stored at −80°C. The protein concentration was determined by spectrophotometry at 280 nm (Nanodrop).

### Mosquito salivation

Fifteen days after their blood-meal, RVFV-exposed mosquitoes were anesthetized at 4°C, legs and wings were sectioned and bodies were placed on a double-sided tape fixed on a glass slide. The proboscis was inserted manually into a 10 µL-cone filled with 5 µL of filtered 1X PBS or DMEM+Glutamax containing 2% FBS. The cone content was collected 45 min later and the virus titer in the solution was determined by plaque assay.

### Infection of mice with ZH548 strain and dissection of organs

Mice were anesthetized intraperitoneally with a mixture ketamine/xylazine consisting of 2 mL of 2% Rompun (Bayer), 4 ml of Imalgene 1000 (Merial), 4 ml of sterile water (Gibco) and 2 mL of 1X PBS (Gibco). “Pathogen-free” male mice C57BL/6NRj (Janvier) aged four weeks and weighing 15–20 g each, were infected in a BSL3 animal facility by intraperitoneal or intradermal route in the absence or presence of either mosquito SGE (one SG pair per inoculums = SGP: 2 µl in 20 µl) or non-infected mosquito bites, or they were infected directly by bites from infected mosquitoes. Selected mice were euthanized five days after infection and the following organs were harvested without any perfusion: brain, liver, spleen, stomach, small and large intestine, pancreas, bladder, heart, lungs, thymus, lymph nodes and salivary glands. Brains were divided into two parts: cerebellum and brain hemispheres (including olfactory bulbs). For virus titration, large organs were cut into pieces of ∼30 mg, whereas small organs like lymph nodes, salivary glands and thymus were kept whole and frozen at −80°C. Samples were then homogenized either in Trizol or in DMEM. Supernatants were collected after centrifugation.

### Histology of mice organs

For these samples, mice were sacrificed 5 days after infection and perfused with 4% formalin. The organs removed were kept in a freshly prepared solution of formalin. The fixed tissues were embedded in paraffin, cut into 3-µm sections thick, and stained with hematoxylin and eosin (H & E).

### Virus titration by plaque assay on E6 cells (Vero)

RVFV-containing samples were titrated on E6 cells by the plaque assay method. Cell counts were performed on KOVA slides. E6 cells were grown in DMEM+Glutamax (Dulbecco) containing 10% decomplemented FBS, 10 U/mL penicillin and 10 µg/mL streptomycin in 6-well plates containing 10^6^ cells per mL for plaque assays. Tenfold serial dilutions of each sample to be titrated were prepared in DMEM medium containing 2% FBS, 10 U/mL penicillin and 10 µg/mL streptomycin. 300 µL of inoculum dilution was deposited in each well of a 6-well plate and incubated with for 1 hr at 37°C in a CO_2_ incubator. Then, 4 mL of agar (culture medium containing 2% FBS and 2% agarose) were deposited in each well and incubated for three days. The plaques were then revealed with a 0.2% solution of crystal violet containing 3.7% formaldehyde and 20% ethanol.

### Detection of anti-RVFV antibodies in mice

For the detection of anti-RVF antibodies in mouse sera, we used a microsphere immunoassay in which a purified recombinant RVF N antigen was covalently associated to color-coded microbeads (unpublished data). Captured anti-RVF antibodies on coupled microspheres were detected using biotinylated anti-mouse IgG and phycoerythrin-conjugated streptavidin by FACS analysis.

### Titration of viral RNAs by qRT-PCR

We used the Power SYBR Green RNA-to-Ct One-Step Kit (Applied Biosystems, Carlsbad, California) according to the manufacturer's protocol. It allowed amplifying a 108 bp sequence located between nucleotide 1485 and nucleotide 1593 of the M segment of RVFV. The primers selected were as follows: upper 5′-CATGGATTGGTTGTCCGATCA-3′ and lower 5′-TGAGTGTAATCTCGGTGGAAGGA-3′. Each sample was analyzed in duplicate against a standard curve produced from a specific concentration range of synthetic RNA. We amplified the samples on an Applied Biosystems 7500 instrument using the following PCR program: a reverse transcriptase (RT) step for 30 min at 50°C; inactivation of the RT enzyme and activation of DNA polymerase for 10 min at 95°C; 40 PCR cycles of 15 sec at 95°C and 1 min at 60°C (annealing temperature of primers), during which fluorescence data is collected; and finally, 20 sec at 95°C with ramping 19 min 59 sec for melting curves.

### Statistical tests used

Results were compared using two nonparametric statistical tests: Kruskal-Wallis and Mann-Whitney. The median day of death was calculated for each condition and results were compared using Kruskal-Wallis and Mann-Whitney statistical tests.

## Results

### Comparison of virus distribution between intraperitoneal and intradermal injection of RVFV

We first selected an optimal mouse strain for our experimental infection model. For this purpose, we infected six C57BL/6 and six DBA-1 male mice by the intradermal route with RVFV and found that the survival curves for these two strains differed significantly. Whereas DBA-1 mice started to die at four days after infection (D4), C57BL/6 mice started to die at seven days after infection (D7). Moreover, whereas neurological symptoms (such as hind limb paralysis) occurred in C57BL/6 mice, no such problems were observed in DBA-1 mice (data not shown). Therefore, we chose the C57BL/6 genetic background for our RVFV infection model.

**Figure 1:**
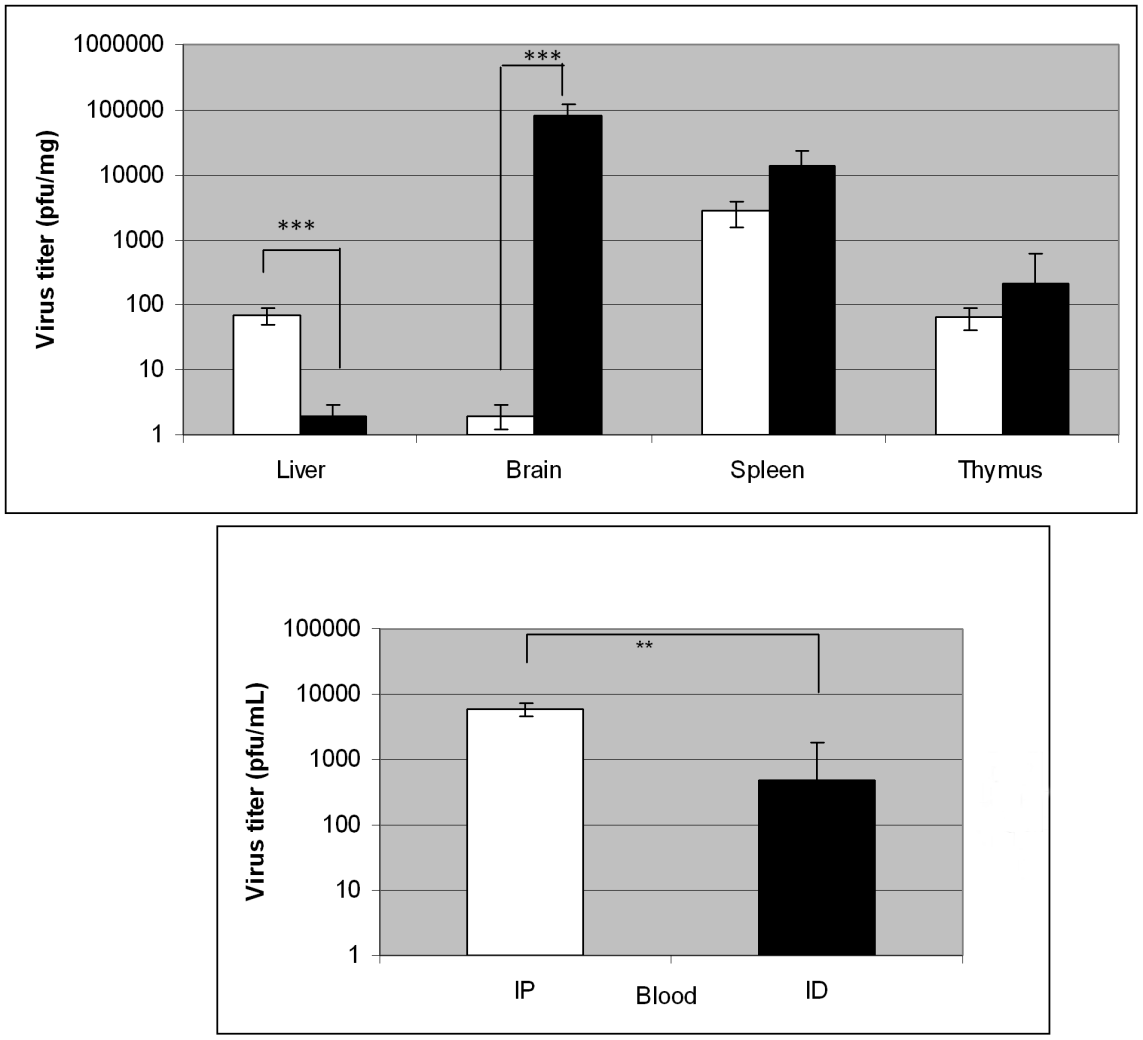
Comparison of RVFV dissemination at D6 in various organs for the two routes of injection. Six C57Bl/6 mice were infected ID or IP with 10^3^ pfu, sacrificed at D6 post-infection and the viral titers were determined by plaque assay. The white bars correspond to the IP route and black bars to the ID route. Data are from 3 independent experiments. * indicates significant differences in viral titers between the two sets of data as determined by Mann-Withney test (** p<0.01); *** p<0.001).

We next compared the mortality rates and RVFV tissue distributions in mice infected by two different routes of injection: the intraperitoneal (IP) and intradermal (ID) routes. The kinetics of infection was slower with the ID route, and a delayed mortality of two days was observed between the two routes of injection (Figure S1). At D3, no significant differences in viremia were found between the two routes of injection. However, at D6, viremia remained at a plateau level of 10^4^ pfu/mL in animals inoculated via IP injection whereas virus titers significantly decreased between D3 and D6 in ID injected mice (Figure 1). Moreover, high virus titers were found in the brain of mice infected by ID injection but not in the liver whereas high titers were found in the liver of mice infected by IP at D6 but not in their brain (Figure 1). In agreement with these findings, ID-infected mice presented neurological symptoms. Since ID infection more closely mimics natural infection by the vector, all subsequent infections were performed by this route.

### Determination of optimal concentration of virus for mice infection

**Figure 2:**
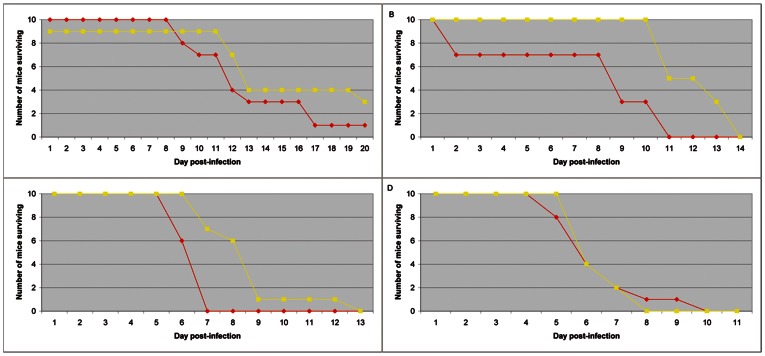
Survival curves of mice. Ten C57Bl/6 mice per group were injected with several doses of RVFV ranging from 10 to 10^4^ pfu/mouse (respectively A to D), in the absence (yellow curve) or presence of 1 SGP (red curve).

To determine whether *Ae. aegypti* mosquito saliva has a role in potentiating RVFV infection, we infected mice ID with doses of virus between 10 and 10^4^ pfu/mouse, with or without SGE from uninfected mosquitoes and calculated the median day of death of the animals for each condition. At the lower dose, not all mice died (Figure 2) and 66% of the mice surviving did not present any anti-RVFV antibodies (data not shown). However, in presence of saliva, all mice but one died (Figure 2) and the surviving mouse presented anti-RVFV antibodies. The effects of SGE on mortality of infected mice were identified at the lower virus doses of 10 to 10^3^ pfu/mouse (Figure 2). Median day of death calculation indicated a significant difference between virus and virus+SGE for injection of 10^2^ and 10^3^ pfu/ml (p = 0.01 and p = 0.002 respectively) (Figure S2). At higher RVFV doses, the effect of SGE on mortality rate was not significant (p>0.05). The weight of the infected mice also decreased as the infections proceeded (data not shown). From these results, we selected 10^3^ pfu/mouse as the reference dose for studying RVFV distribution in mice in the presence and absence of SGE.

In addition, we found that the effect of the SGE was not restricted to *Ae. aegypti* mosquitoes as *Ae. vexans* SGE also increased RVFV virulence (Figure S3). Median day of death calculation indicated a significant difference between virus and virus+SGE (p = 0.006) and virus+saliva (p = 0.01). However, we did not observe any difference between virus+SGE and virus+saliva (p = 0.42). Interestingly, we did not observe any effect on mice survival when we injected *Culex pipiens pipiens* SGE (data not shown).

### Distribution of RVFV in the organs of C57BL/6 mice following ID infection with 10^3^ pfu/mouse with and without SGE

**Figure 3:**
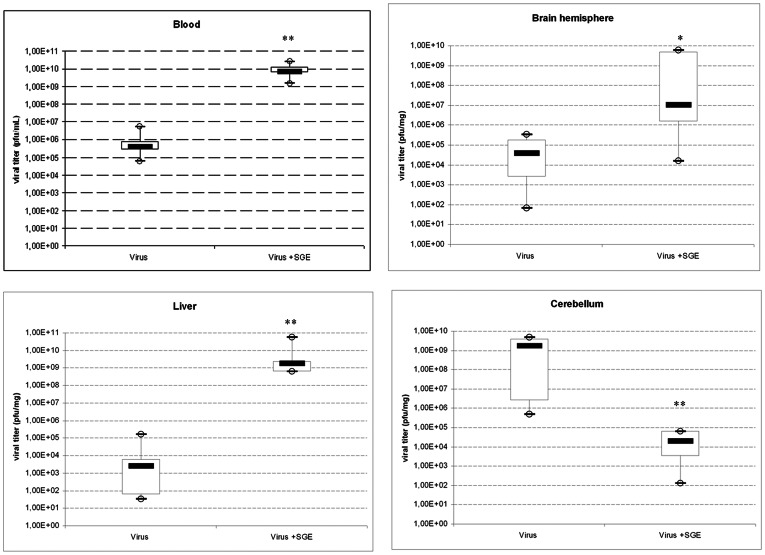
Viral titers of major target organs at D5 post-infection. Three lots of **5 C57Bl/6 mice** were infected by ID injection of 10^3^ pfu RVFV with or without 1 SGP. RVFV titer was determined by plaque assay on E6 cells at D5 post-infection. Results are presented as box-and-whisker, indicating inside the box the median titer value and the bottom and top of the box being the 25th and 75th percentile. Whiskers correspond to the lowest and largest values of the titers. Mann-Withney test was employed to analyze the difference between sets of data for each organ. * p<0.05; ** p<0.01.

We infected C57BL/6 mice with ID injections of virus (10^3^ pfu/mouse) in the presence or absence of SGE and followed the distribution of the virus in the blood and in various organs. The organs were not perfused prior collection. We sacrificed the mice at D5 because in Figure 2 infected mice died during the night between D5 and D6. Viremia levels were very high in the infected mice, and high virus titers were also found in the liver, brain and cerebellum (Figure 3), lymphoid organs (spleen, thymus and lymph nodes) (Figure S4), as well as in heart, kidneys, and lungs (Figure S5). Low virus titers were found in the eyes, jejunum and ileum (less than 10^3^ pfu/mL), whereas the intestine, stomach, ceacum, colon and gallbladder contained no measurable titers.

In the presence of SGE, virus titers were significantly increased (almost 10^4^ fold) than those produced in the absence of SGE, in the brain cortex (p = 0.024), liver (p = 0.004) and blood (p = 0.004) (Figure 3). The virus titers in the cerebellum exhibited an opposite trend compared to the titers in the brain cortex. Indeed, in this organ, virus titers of animals infected in the presence of SGE were lower (median value of 10^4^ fold) than those of animals infected without SGE (p = 0.004).

We then analyzed the virus titers in the lymphoid organs of mice infected in the presence and in the absence of SGE. The virus titers in the inguinal lymph nodes (p = 0.03), spleen (p = 0.004) and thymus (p = 0.007) of mice infected in the presence of SGE were significantly higher than those of mice infected in the absence of SGE (Figure S4). Virus titers in the lungs (p = 0.004), kidneys (p = 0.005), bladder (p = 0.004) and heart (p = 0.01) of mice infected in the presence of SGE were significantly higher (10^2^ fold increase) compared to the titers found in these tissues of mice infected without SGE (Figure S5). Virus titers in the pancreas exhibited a pattern similar to that observed in the cerebellum, as these titers were significantly lower in the presence of SGE (p = 0.016) than in absence of SGE. In contrast, the addition of SGE to the viral inoculum did not lead to any significant differences in the virus titers in the mesenteric lymph nodes (ML), aortic lymph nodes (AL), popliteal lymph nodes (PL) or salivary glands (data not shown). These results correlated well with the RNA quantification results we obtained from RT-qPCR analysis of each organ (data not shown).

### Effect of SGE on the blood cell counts of infected mice

Following RVFV infection of mice with or without SGE, we found that several blood parameters were altered in infected mice compared to uninfected ones. These changes included significantly lower numbers of white blood cells and platelets (Table 1).

**Table 1:** Distribution of different cells in the blood of infected mice at 5 DPI.

Cells	Units	Non-infected mice	mice+RVFV	mice_+_RVFV/SGE
**White blood cells**	**10^3^/mm^3^**	**3.6±0.2** abc	**2.4±0.1** abc	**2±0.3** abc
Red blood cells	10^6^/mm^3^	6.7±0.4	6.8±0.3	7.4±1
hemoglobin	g/dl	10.8±0.6^c^	10.7±0.5	11.9±1^c^
hematocrit	%	36.5±1.5^c^	36,9±1.6	41.2±3.6^c^
**platelets**	**10^3^/mm^3^**	**1099.3±186** **2±0.3** abc	**704±94** **2±0.3** abc	**517.2±162** **2±0.3** abc
lymphocytes	%	87.4±0.8^ac^	64.4±3.8^a^	62.3±20.4^c^
monocytes	%	3.4±0.2^ac^	9.4±1.7^a^	7.8±2.1^c^
granulocytes	%	9.2±0.9^ac^	26.2±2.4^a^	29.8±18.7^c^

a,b,c The statistical significance of different comparisons (p<0.05) are represented by letters: a) uninfected mice vs. ZH-infected mice; b) RVFV-infected mice vs. RVFV+SGE infected mice; and c) healthy mice vs. RVFV+SGE infected mice. Ten mice were tested in each group.

The significant leukopenia observed in infected mice was associated with changes in the white blood cell count, with proportionally higher numbers of granulocytes and monocytes and lower numbers of lymphocytes compared to those in uninfected mice (Table 1). A 50% decrease of platelets and white blood cell counts was observed in presence of SGE in the inoculation (Table 1).

### Histological analysis of liver from RVFV-infected C57BL/6 mice

**Figure 4:**
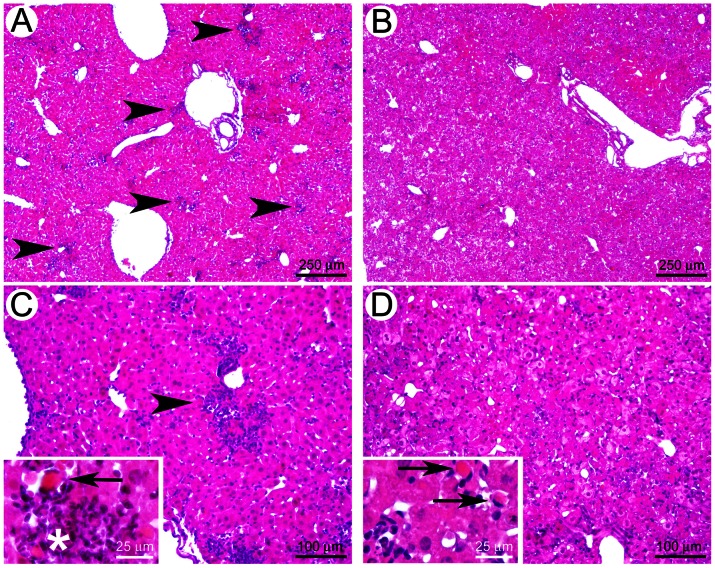
Histological sections of liver at D5 post-infection. Five C57Bl/6 mice were injected with RVFV at 10^3^ pfu/mouse. Images A and C correspond to the livers of mice infected in the presence of 1 SGP and viewed at 4X and 10X magnification, respectively (inset in C is 40X). The arrow head in A and C identify inflammatory foci. The arrow in the inset of C identifies a necrotic hepatocyte, and the asterisk identifies neutrophil infiltration. Panel B and D correspond to the livers of mice infected in the absence of SGE at 4X and 10X magnification, respectively. The arrows in the inset in D show a necrotic hepatocyte foci.

To better understand the physiology of RVFV infection, we conducted histological analysis of the liver of infected mice and found significant differences between the livers of mice infected in the presence or absence of SGE (Figure 4). Indeed, mice infected in the presence of SGE exhibited signs of multifocal hepatitis (Panels A and C). Inflammatory foci were randomly distributed in the liver parenchyma (arrowheads in Panels A and C). These foci were characterized by prominent neutrophil infiltrations (asterisk in Panel C insert) that were associated with fewer numbers of lymphocytes. Necrotic hepatocytes, with acidophilic cytoplasm and a highly condensed basophilic nucleus (pyknosis) or a fragmented nucleus (karyorrhexis) were identified within the inflammatory foci (arrow in Panel C insert). The profile of the liver lesions in mice infected in the absence of SGE (Figure 4; Panels B and D) was very different from that of mice infected in the presence of SGE. Three out of four mice exhibited hepatic necroses with very few inflammatory foci (Panel B). These necrosis foci were randomly located within the parenchyma (arrows in Panel D insert) and were associated with few inflammatory cells (Panel D insert).

### Effects of exposing mice to non-infected and RVFV-exposed *Ae. aegypti* mosquito bites

We collected saliva from RVFV-exposed mosquitoes to estimate the concentration of virus injected during a bite. These results showed mosquitoes may inject approximately 50±20 pfu in each bite, and that more than 50% of the mosquitoes had been infected after an artificial blood-meal.

**Figure 5:**
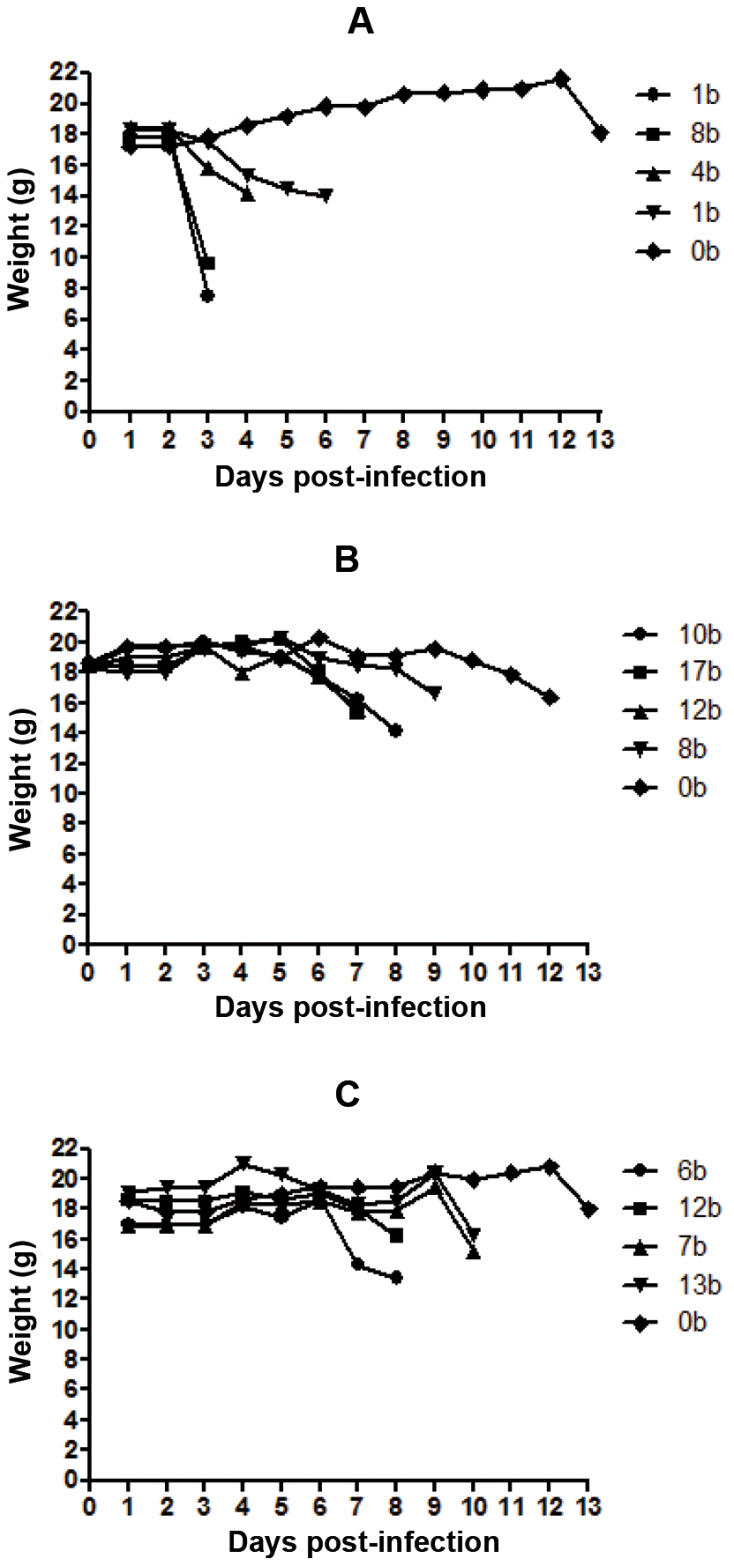
Weight curves of mice infected by ID and bitten by non-infected mosquitoes. Three lots (A, B and C) of 5 C57Bl/6 anesthetized mice were used for each experiment. They were injected ID with 50 pfu of RVFV before being exposed to the bites of 20 mosquitoes contained in cardboard boxes. After 15 minutes, blood-fed mosquitoes were collected and counted. After exposure, the weight of each mouse was recorded every day. Each curve corresponds to one mouse and the number of non-infected bites is indicated in the legend on the figure. Death occurred at the end of each curve.

To this point, our experiments were performed with SGE, which contains a mixture of salivary and housekeeping proteins. To determine whether the unique components of saliva triggered the potentiating effect on RVFV virulence, we allowed ID-infected mice to be bitten by non-infected mosquitoes. We inoculated C57BL/6 male mice with RVFV (50 pfu/mouse) by ID injection and exposed the mice to non-infected mosquito bites in the area of the ID infection. The number of blood-fed mosquitoes was determined. The weight changes of the mice were followed for 14 days thereafter. The weight curves of the infected mice corroborated with our previous results (Figure 5). In the absence of mosquito bites, mice survived for at least 11 days and died between 13 and 14 days post infection. If infection was accompanied by non-infected bites, time to death was shortened. We however did not find any clear correlation between the number of bites and the time to death.

**Figure 6:**
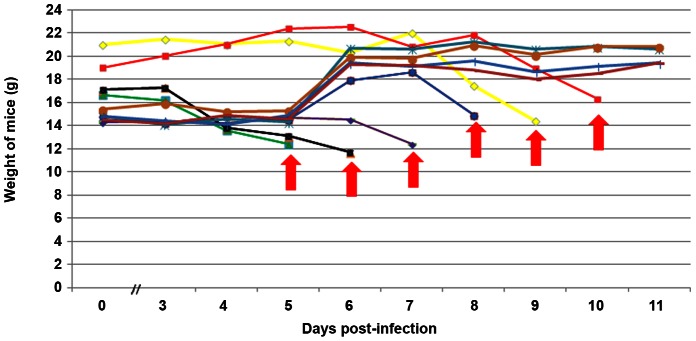
Weight curves of mice bitten by RVFV-exposed *Aedes aegypti*. Ten C57Bl/6 mice were anesthetized and exposed to bites of mosquitoes collected at D19 after virus exposure. The blue and violet curves correspond to mice bitten by 6 mosquitoes (6 of 6 and 4 of 6 mosquitoes were infected respectively). The yellow curve represents a mouse bitten by 8 mosquitoes (3 of 8 mosquitoes were infected). The red curve corresponds to a mouse bitten by 3 (3 of 3 mosquitoes were infected). The black curve corresponds to 7 mosquito bites (4 of 7 mosquitoes were infected) and the green curve corresponds to 9 mosquito bites (3 of 9 mosquitoes were infected). The red arrow indicates the death of the mouse. Two other mice were bitten by 6 mosquitoes (6 of 6 and 4 of 6 mosquitoes were infected respectively) and did not die after 11 days of observation (dark red and light blue curves). Mosquitoes did not feed on 2 mice (brown and turquoise curves).

In a second series of experiments, mice were bitten by RVFV-exposed mosquitoes collected on D16 or on D19 after infected blood meal. Blood-fed mosquitoes were collected and their viral loads were determined. The weight curves for the mice bitten by mosquitoes at D16 after virus exposure were followed. Three out of five mice received 1 or 2 bites from RVFV-exposed mosquitoes. Only one mouse died 13 days after receiving four mosquito bites (for which two out of four mosquitoes were infected), while the other mice survived until day 14 (data not shown). The experiment was repeated with mice bitten by D19 RVFV-exposed mosquitoes (Figure 6). As before, the numbers of blood-fed mosquitoes were counted and their viral loads were determined. Mice received up to 9 bites and 3 to 6 of these bites were from infected mosquitoes. Two mice having received bites from infected mosquitoes did not die during the time of experiment (11 days). Their weight did not decrease. Mosquitoes did not feed on two mice. For the other 6 mice, death was observed from day 5 to day 10 post-feeding. The time to death did not depend on the number of blood-fed mosquitoes collected on each mouse and is more probably related to the amount of virus injected during the probing phase and to the number of uninfected bites. This amount seems highly variable in our experiment. Indeed, we were not able to identify mosquitoes that could have probed, and thereby injected virus, without taking any blood meal.

## Discussion

RVFV is primarily transmitted by mosquito bites and, to a lesser extent, by direct contact with infected animals, mainly sheep and goats, as reported during an RVF epidemic in southwestern Saudi Arabia [7]. However, many studies describing the pathogenesis of this virus have been conducted without considering this natural way of transmission. Indeed, the route of virus inoculation and the presence of components from the vector saliva are likely to have consequences on the immune response that is eventually developed by the host in response to the pathogen. In fact, several studies have shown that the saliva of arthropod vectors transmitting infectious diseases can play a crucial role in the ability of the vector to transmit the pathogen [15].

The mouse strain used in any model of RVFV infection is an important factor that should be carefully considered. Several different genetic strains of mice have been used previously: BALB/cByJ, C57BL/6, 129/Sv/Pas, and MBT/Pas [20]. The BALB/cByJ, and C57BL/6 strains were found to be the least susceptible to RVFV infection. In our study, DBA-1 mice were more sensitive to the virus than C57BL/6 even though they did not exhibit any neurological symptoms. The C57BL/6 strain experienced hepatic infection as well as neurologic symptoms. These mice are therefore good models to study the most severe forms of RVF in humans, and allow the study of neuropathogenesis and progression of the virus from the periphery to the central nervous system following intradermic inoculation.

A number of different studies aimed at defining the pathogenesis of RVFV in animals have employed IP, intranasal and subcutaneous inoculations [4], [21]. Indeed, exposing mice to aerosols containing RVFV can cause infection [22] whereas other routes of exposure induce delayed death [21]. In our study, the IP and ID routes of injection led to different patterns of virus dissemination. The brain and liver were the main targets of the virus after ID and IP infection, respectively. Viremia was maintained longer after IP infection whereas survival was shorter compared to ID infection. This result showed that the route of infection is a key determinant for infection.

First, after ID injection, we found the virus in many organs. High virus titers were found in the liver and in the blood early after infection at D3, whereas at D6, high virus titers were found in the brain, while the viremia has decreased. In agreement, mice presented neurological symptoms at D6, characterized by compulsive or uncoordinated movements, and/or paralysis, and they also had discolored livers presenting hemorrhagic lesions. Our observations correlated well with other studies that showed that this virus causes fulminant hepatitis [7] or meningoencephalitis [23] in humans. We found other organs to be less infected, including the heart, lungs, pancreas and kidneys, which was reported previously [24]. Mice salivary glands were found to be significantly infected, raising the question of the infectivity of saliva. Viral antigens have also been detected in odontogenic and gingival epithelia [24]. In addition, we detected virus in the primary and secondary lymphoid organs and the lymphocyte numbers were lower in infected animals compared to controls. These changes could be explained by lymphocyte apoptosis in lymphoid organs (thymus, spleen and lymph nodes), which was also demonstrated in BALBc mice subcutaneously infected with another RVFV strain (ZH501) [24].

We also observed changes in other blood count parameters like the number of thrombocytes (platelets), granulocytes and monocytes. In general, a decrease in circulating platelet number may be caused by decreased or ineffective bone marrow production, increased intramedullary destruction (hemophagocytic syndrome), increased peripheral destruction (immune-mediated or non–immune-mediated mechanisms), altered distribution of circulating cells (splenic consumption or endothelial sequestration), or decreased cellular life span. Bone marrow was found to be infected in our study (data not shown), and lower numbers of myeloid cells in the spleen and bone marrow in RVFV infection have been reported previously [24]. On the other hand, in patients with dengue hemorrhagic fever, although dengue virus-induced bone marrow suppression was shown to decrease platelet synthesis, an immune mechanism of thrombocytopenia caused by increased platelet destruction appeared to be also active [25], [26], [27], [28]. Granulocytes and monocytes numbers were higher in the blood of infected animal (Table 1). Three types of granulocytes are present in peripheral blood: neutrophils, eosinophils and basophils. The count of eosinophils was found to vary as function of Rift Valley fever disease progression in mice: it first decreased at the beginning and then increased before death [29], which could explain our findings. Granulocytes were also found to be important target cells for RVFV infection [21] and thereby represent a site of viral replication to infect other cells or organs. Monocyte numbers also increase in many other vectorial infectious diseases such as West Nile, dengue and malaria [24], [25], [26]. Similar changes in blood cell counts including lymphopenia and thrombopenia were reported for the Saudi Arabian epidemics of 2000 [7].

We investigated the role of vector salivary components in RVFV infection. Potentiation of virus transmission and/or pathogenicity in the presence of vector saliva has previously been described in vector/pathogen/host interactions [14], [15], [30]. Some of the many salivary proteins co-injected during a vector bite cause immunomodulatory effects on the host. These may include the induction of a Th2 response and the inhibition of Th1 pro-inflammatory cytokines [15], [31]. In addition, it has been shown *in vivo* that *Aedes* mosquito bites are likely to significantly reduce T cell recruitment [16]. We tested the saliva of two *Aedes* species: *Ae. vexans*, which is an important RVFV vector in Africa and in the Arabic peninsula [32], [33], [34], [35]; and *Ae. aegypti*, which exhibits good vector competence for the virus as shown in our study as well as in others [11], [36] and whose genome has been sequenced. Early death was observed in the groups of mice co-injected with both *Aedes* SGE. In addition, the survival curves obtained for RVFV-infected mice exposed to the bites of mosquitoes corroborated those obtained with co-injected SGE and confirmed that both *Ae. aegypti* and *vexans* saliva potentiates RVFV pathogenicity. These results are comparable to those reported for mice with West Nile virus mixed with mosquito saliva [31]. Interestingly, although *Culex pipiens* was found to be competent to transmit RVFV [37], we did not observe any increase of RVFV pathogenenicity in presence of salivary gland extracts from this species.

We determined the effects of SGE and saliva on RVFV virulence and distribution for several organs and included histological analyses of the liver. For most organs, including liver, the brain cortex, kidneys, lungs, heart, bladder, spleen, thymus and lymph nodes, virus titers were significantly higher if SGE was included in the inoculum, in agreement, with previous studies where saliva was shown to increase the invasion of neural tissues by West Nile virus and produced higher virus titers in the brain [31]. An SGE-mediated decrease in antiviral activity at the site of inoculation might promote viral replication and infection of different cell types [20], [21], [38], thereby increasing virus production in several organs and causing specific histological lesions, as observed in the liver. This is consistent with what Schneider and Higgs observed in mice infected with West Nile virus in presence of mosquito bites [31]. Early after virus inoculation, they did not observe any difference in the viral titers measured in various organs in presence or absence of saliva whereas after 7 days of infection, higher titers were observed after mosquito bites. We cannot exclude however a modification of the kinetics of virus replication and dissemination in the various tissues in the presence of saliva. Interestingly, and while the viremia is significantly increased, lower virus titers were found in the pancreas and cerebellum in presence of SGE, showing that saliva may also affect virus dissemination. With respect to the brain, our results suggest that saliva might modify the kinetics and/or the extent of invasion of specific regions. The modalities of infection of the central nervous system by RVFV are still poorly understood. Neurons and glial cells were found positive for RVFV throughout the central nervous system of infected calves [39]. Gray et al. [29] showed that the brains of RVFV ZH501 infected mice were essentially normal throughout the course of the study despite evidence of a high viral titer and significantly increased inflammatory cytokine concentrations in the brain tissue of some studied animals. The outcome of our study may suggest either that the presence of saliva at the site of inoculation may favour different ways of brain invasion or that the kinetics of infection is increased and that the cerebellum was first invaded and already partly cured at the moment of sample harvesting while the virus was spreading towards the brain cortex.

Since a direct effect of saliva on the brain is unlikely, we propose that modulation of the early immune and inflammatory responses at the site of virus injection may, in turn, modulate the permeability of the blood-brain barrier, allowing virus titers in the brain to be significantly higher. Further studies on this matter are currently underway and preliminary experiments are in favor of an increase of the vascular permeability of the blood brain barrier in presence of saliva. We suggest that intermediate elements like TLR3 and IL6 might be involved in this effect. Actually, West Nile virus, by activating TLR3 (toll-like receptor 3) [40], and allowing TNFα secretion, was proposed to increase blood-brain barrier permeability. Moreover, it was shown that IL-6 played an important role in increasing brain permeability in a model of bacterial meningitis [41], [42]. Similar mechanisms might occur in RVFV infections in the presence of saliva.

Our histological analysis of infected liver showed that mice infected in the presence of SGE developed multifocal hepatitis with inflammatory foci that were randomly distributed in the hepatic parenchyma. This was also accompanied by a massive recruitment of neutrophils and lymphocytes in the liver parenchyma. CD4+ and CD8+ lymphocytes and cytokines, including TGF-β, TNF-α and IFN-γ were shown to be involved in the hepatic pathogenesis of yellow fever virus infection in combination with a direct cytopathic effect of the virus [43]. The early modulation of the innate response in the dermis caused by mosquito bites probably induces a dysregulation of the immune system and triggers the different pathologic effects observed in absence and presence of the mosquito saliva.

Exposure of inoculated mice to mosquito bites confirmed that saliva components have a potentiating effect on RVFV infection. Indeed, we observed early death in mice infected by ID and bitten by uninfected mosquitoes although a clear correlation between the number of engorged mosquitoes and the time of death could not be established. This is probably explained by the time of probing that differs between mosquitoes and the length of the probing time conditioned the amount of saliva injected in the dermis.

The next step was to compare infection by an infected mosquito to infection by ID. Death was observed as early as day 5 post-infection, a delay which is comparable to that of mice infected ID with 10^3^ pfu in the presence of SGE. This observation shows that mosquitoes may inject more than 50 pfu in agreement with the detection of a discrepancy between the titers obtained by salivation and those determined in vivo [44]. Our results also showed that the bites of non-infected mosquitoes may potentiate infection caused by the bites of infected mosquitoes. It is important to note that although the number of infected mosquitoes in nature is relatively low, the number of uninfected bites is much higher. Thus, constant local stimulation with saliva may have the potential to modulate the impact of RVFV infection [45].

In conclusion, we have clearly demonstrated an overall potentiating effect of mosquito saliva on RVFV infection. Both *Aedes aegypti* and *Aedes vexans* saliva are able to decrease the survival of RVFV-infected mice. The impact of saliva components on the innate immune response at the site of bite certainly explains the facilitation observed, either by increasing the kinetics of distribution of the virus or by altering this distribution through differential targeted organs. The identification of salivary proteins involved in the facilitation of infection and determination of their mode of action could help develop new approaches for preventive or therapeutic purposes in humans.

## Supporting Information

Figure S1 **Survival curves of mice injected ID or IP with RVFV.** Ten C57Bl/6 mice were infected with 10^3^ pfu of RVFV ZH 548 strain by IP (pink) or by ID (blue) routes. Animals were examined each day.
(TIF)

Figure S2 **Survival of mice infected with RVFV at several doses/mouse with or without** ***Ae. aegypti*** **SGE.** Groups of 10 C57Bl/6 mice were infected by ID, with RVFV+1 SGP. The median day of death was determined for each condition and sets of data were analyzed using Kruskal-Wallis and Mann-Whitney statistical tests.
(TIF)

Figure S3 **Survival of mice infected with RVFV with or without SGE or saliva from** ***Ae. vexans*** **.** Groups of 10 C57Bl/6 mice were infected by ID, with RVFV alone (10^3^ pfu/mouse), with RVFV+1 SGP or with RVFV+non-infected mosquito bites. The median day of death was determined for each condition and sets of data were analyzed using Kruskal-Wallis and Mann-Whitney statistical tests.
(TIF)

Figure S4 **Virus titers of lymphoid organs on post-infection D5.** Three lots of 5 C57Bl/6 mice were infected by ID injection of 10^3^ pfu RVFV with or without 1 SGP. RVFV titer was determined by plaque assay on E6 cells at D5 post-infection. Data are from 3 independent experiments, each performed on five mice. Mann-Withney test was employed to analyze the difference between sets of data for each organ. * p<0.05; ** p<0.01.
(TIF)

Figure S5 **Virus titers of secondary target tissues on post-infection D5.** Three lots of 5 C57Bl/6 mice were infected by ID injection of 10^3^ pfu RVFV with or without 1 SGP. RVFV titer was determined by plaque assay on E6 cells at D5 post-infection. Data are from 3 independent experiments, each performed on five mice. Mann-Withney test was employed to analyze the difference between sets of data for each organ. * p<0.05; ** p<0.01.
(TIF)
