# Supplementary figures and images for: Aedes Mosquito Saliva Modulates Rift Valley Fever Virus Pathogenicity
Source: PLoS Negl Trop Dis. 2013 Jun 13;7(6):e2237. doi: 10.1371/journal.pntd.0002237 (PMC3681724; doi:10.1371/journal.pntd.0002237)

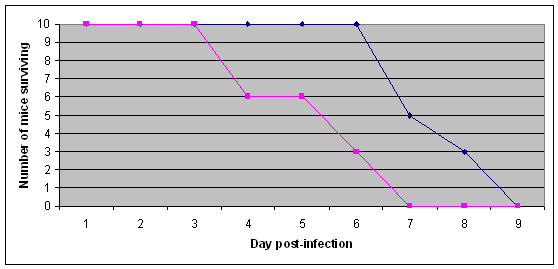

Supplement: Figure S1 — Survival curves of mice injected ID or IP with RVFV. Ten C57Bl/6 mice were infected with 103 pfu of RVFV ZH 548 strain by IP (pink) or by ID (blue) routes. Animals were examined each day. (TIF) [file pntd.0002237.s001.tif]

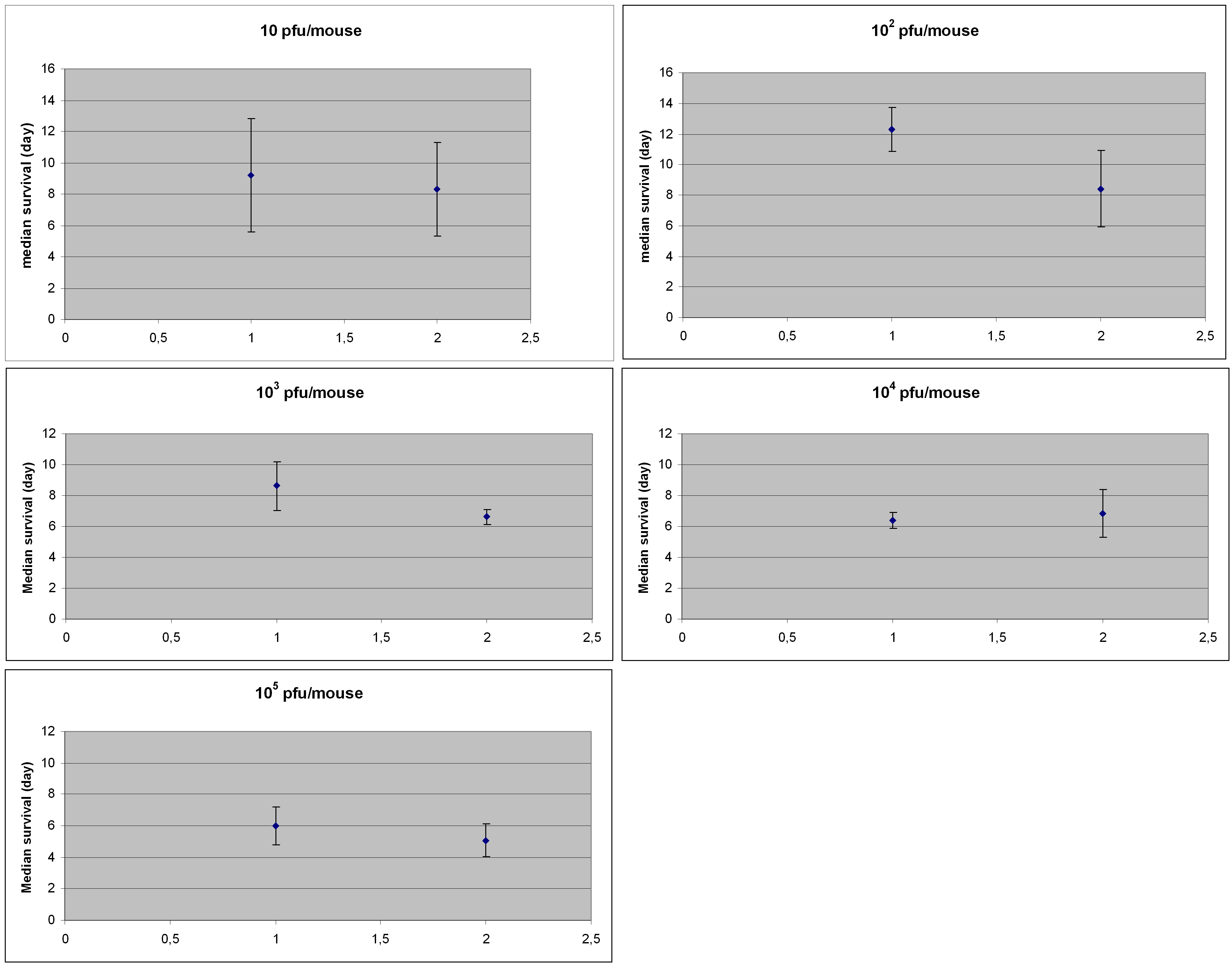

Supplement: Figure S2 — Survival of mice infected with RVFV at several doses/mouse with or without Ae. aegypti SGE. Groups of 10 C57Bl/6 mice were infected by ID, with RVFV+1 SGP. The median day of death was determined for each condition and sets of data were analyzed using Kruskal-Wallis and Mann-Whitney statistical tests. (TIF) [file pntd.0002237.s002.tif]

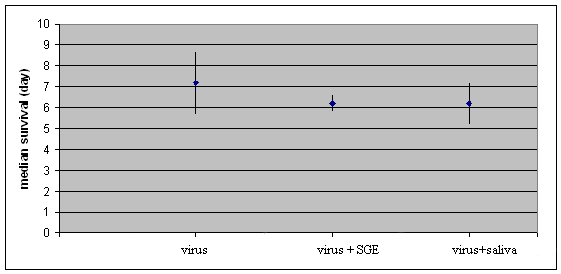

Supplement: Figure S3 — Survival of mice infected with RVFV with or without SGE or saliva from Ae. vexans . Groups of 10 C57Bl/6 mice were infected by ID, with RVFV alone (103 pfu/mouse), with RVFV+1 SGP or with RVFV+non-infected mosquito bites. The median day of death was determined for each condition and sets of data were analyzed using Kruskal-Wallis and Mann-Whitney statistical tests. (TIF) [file pntd.0002237.s003.tif]

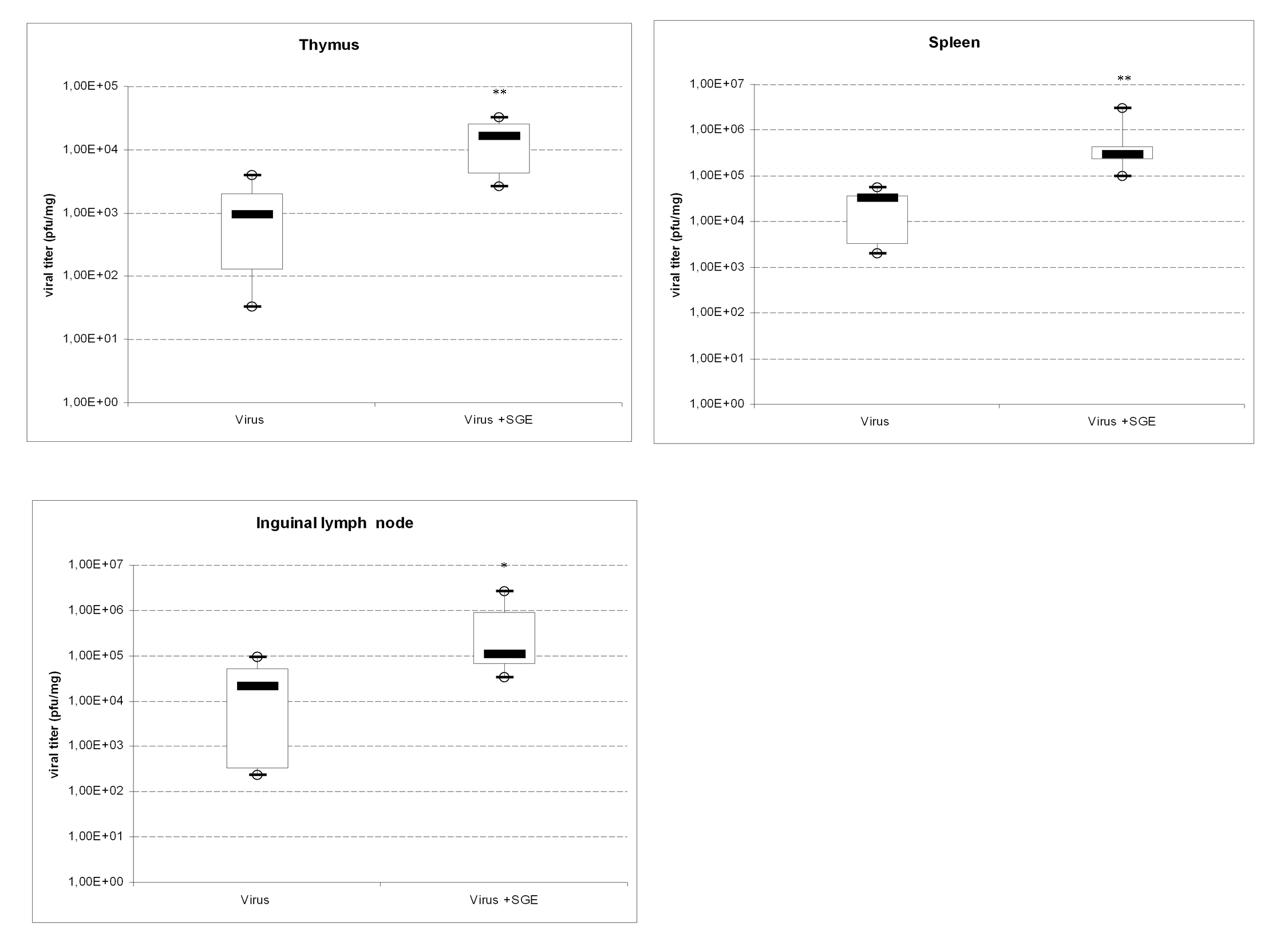

Supplement: Figure S4 — Virus titers of lymphoid organs on post-infection D5. Three lots of 5 C57Bl/6 mice were infected by ID injection of 103 pfu RVFV with or without 1 SGP. RVFV titer was determined by plaque assay on E6 cells at D5 post-infection. Data are from 3 independent experiments, each performed on five mice. Mann-Withney test was employed to analyze the difference between sets of data for each organ. * p<0.05; ** p<0.01. (TIF) [file pntd.0002237.s004.tif]

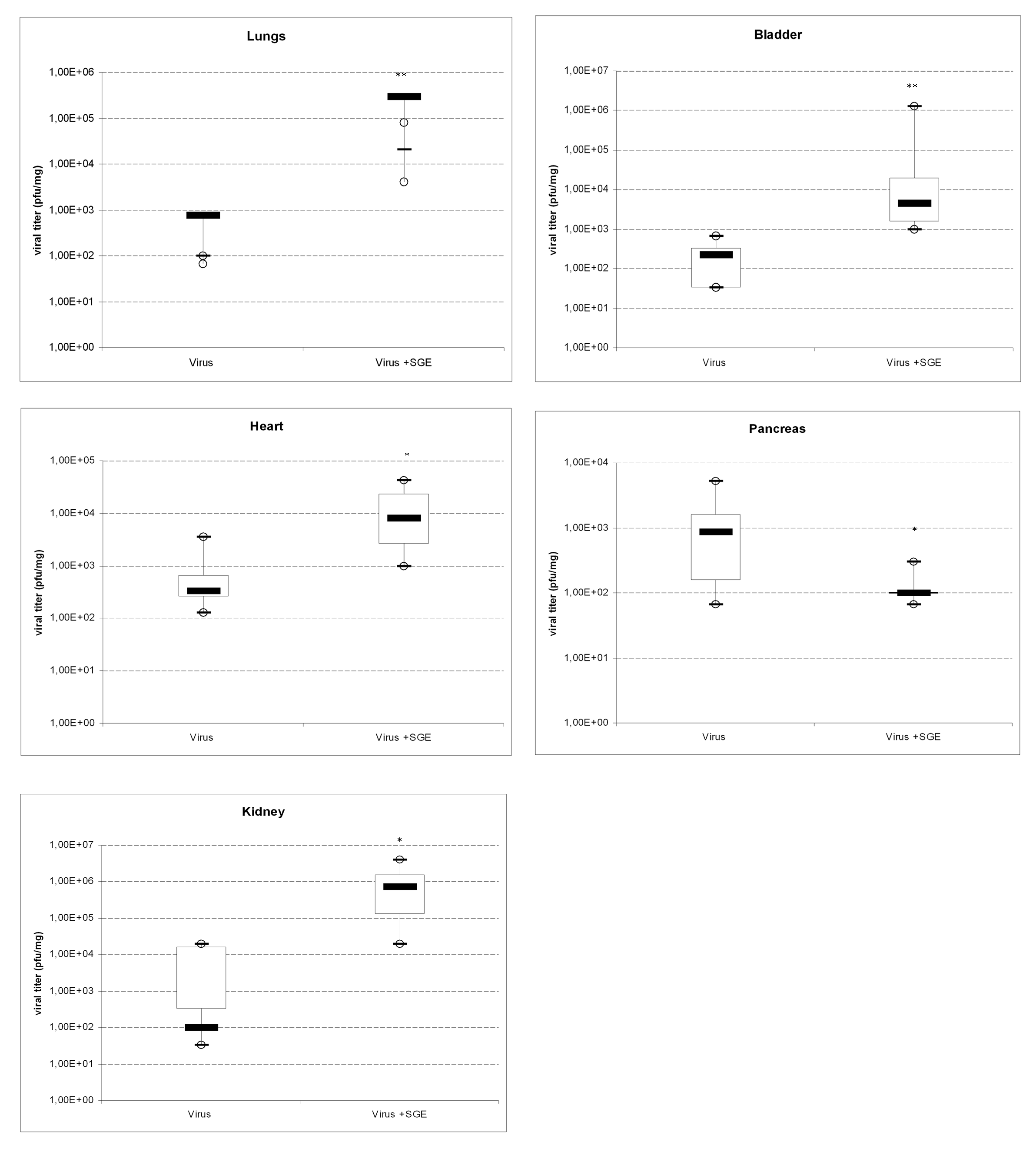

Supplement: Figure S5 — Virus titers of secondary target tissues on post-infection D5. Three lots of 5 C57Bl/6 mice were infected by ID injection of 103 pfu RVFV with or without 1 SGP. RVFV titer was determined by plaque assay on E6 cells at D5 post-infection. Data are from 3 independent experiments, each performed on five mice. Mann-Withney test was employed to analyze the difference between sets of data for each organ. * p<0.05; ** p<0.01. (TIF) [file pntd.0002237.s005.tif]
